# Maintenance and disruption of mucous barrier homeostasis: the critical role of MUC5AC/MUC5B balance in airway diseases

**DOI:** 10.3389/fmed.2026.1777480

**Published:** 2026-05-08

**Authors:** Ying Song, Xiao Wu, Haolin Tian, Zheng Liu

**Affiliations:** Nanjing Hospital of Chinese Medicine Affiliated to Nanjing University of Chinese Medicine, Nanjing, Jiangsu, China

**Keywords:** airways, MUC5AC, MUC5B, mucociliary clearance, mucus

## Abstract

Airway diseases pose a major challenge to global health, with excessive mucus secretion being one of their common pathological cores. As the primary functional components of the airway mucus gel, MUC5AC and MUC5B jointly maintain mucus barrier function under physiological conditions. However, during disease progression, dysregulation of these two mucins becomes a key factor driving disease advancement. Understanding the expression patterns and regulatory mechanisms of MUC5AC and MUC5B in disease states is crucial for elucidating related pathological mechanisms. This review systematically summarizes relevant studies to explore the dynamic changes of these two mucins across multiple airway diseases, aiming to provide new perspectives and potential targets for precision diagnosis and treatment of airway diseases.

## 1 Introduction

Airway mucus, a heterogeneous mixture of water, mucins, proteins, salts, and lipids, is traditionally listed among lung innate defenses owing to its ability to trap and mediate swift removal of inhaled particles/pathogens by mechanical clearance. Effective mucus clearance is essential for respiratory health, as illustrated by the poor prognosis of lung diseases characterized by airway mucus stasis, e.g., cystic fibrosis, primary ciliary dyskinesia, the bronchitis associated with chronic obstructive pulmonary disease, and bronchiectasis (1).

The airway mucus barrier serves as the primary line of defense for maintaining the host defense function of the respiratory tract. The integrity of its structure and function is primarily maintained by the gel-forming mucins MUC5AC and MUC5B (2, 3). This review focuses on the distinct roles and dysregulation of MUC5AC and MUC5B, with emphasis on how restoring their physiological balance may offer novel therapeutic opportunities.

## 2 Methods

This review conducted a comprehensive literature search across major Chinese academic databases (including CNKI and Wanfang Data) and English-language databases (including PubMed, Web of Science, and Embase) to identify all eligible basic and clinical studies examining mucin expression in airway diseases.

**Figure 1:**
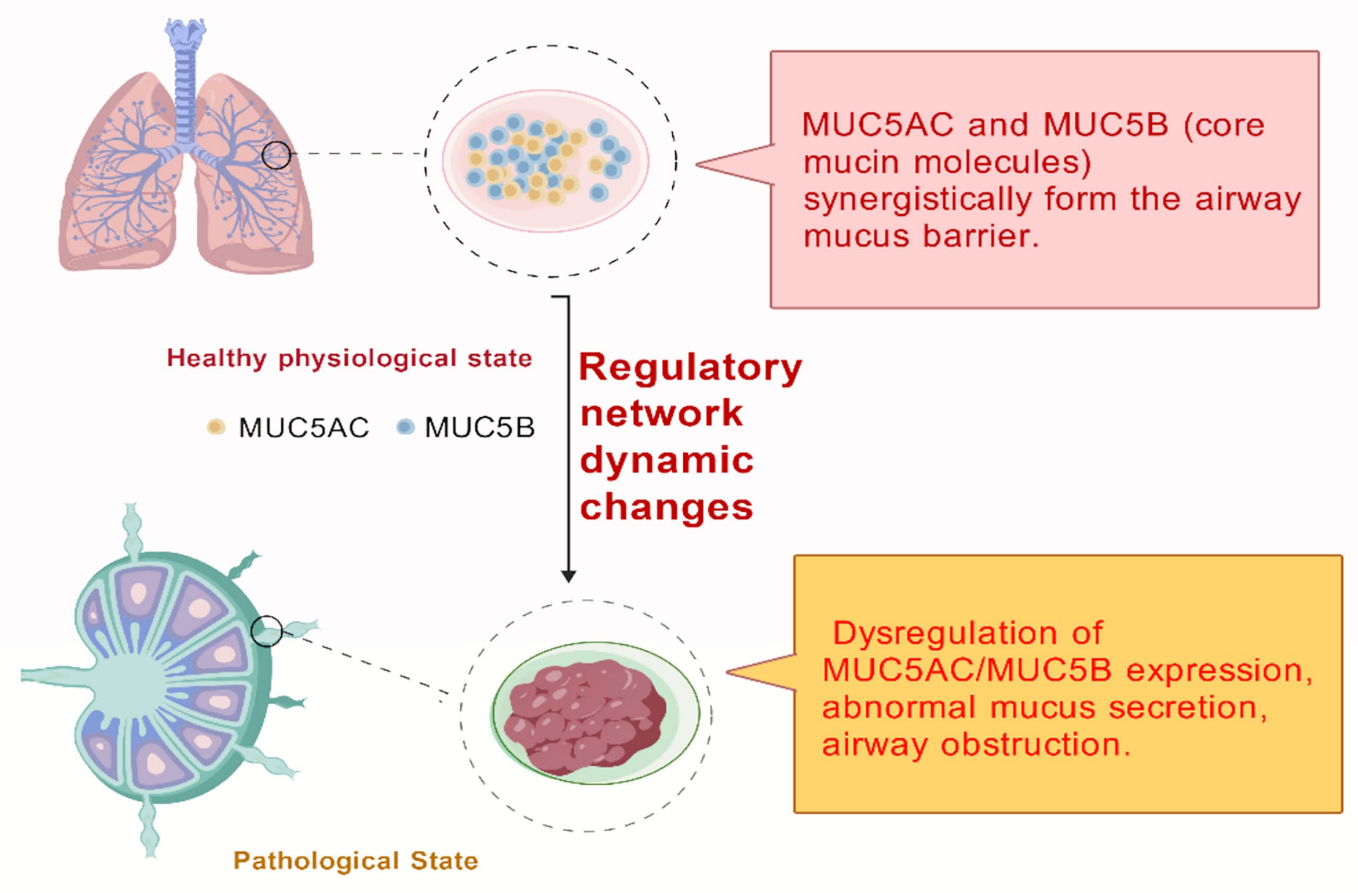
Dynamic balance and functional changes of MUC5AC/MUC5B in physiological and pathological airway states.

The search strategy employed predefined targeted keywords covering mucin, airway diseases, MUC5AC, and MUC5B-related terms. Priority was given to literature published from 2020 to the present to capture the latest research advances, cutting-edge findings, and clinical application trends in this field, ensuring the timeliness and state-of-the-art nature of this review. Subsequently, a comprehensive search was conducted covering all eligible literature to avoid omitting foundational classic studies and key findings, ensuring the comprehensiveness and completeness of the retrieval (Figure 1).

**Figure 2:**
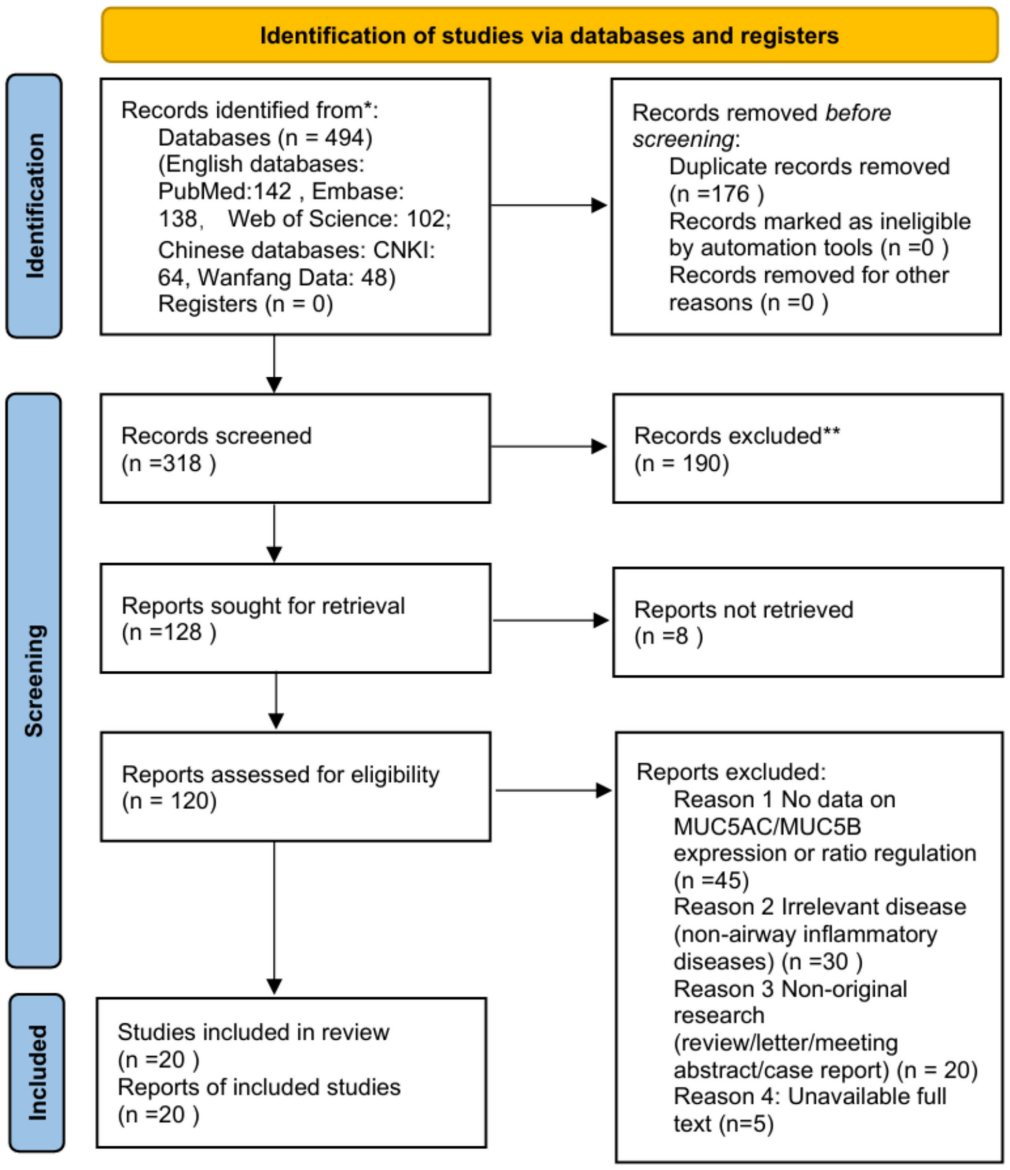
PRISMA 2020 flow diagram for the systematic review of MUC5AC/MUC5B imbalance in airway inflammatory diseases. ^*^Consider, if feasible to do so, reporting the number of records identified from each database or register searched (rather than the total number across all databases/registers). ^**^If automation tools were used, indicate how many records were excluded by a human and how many were excluded by automation tools.

Literature was screened based on explicit inclusion and exclusion criteria (e.g., study type being basic experimental or clinical research, subjects being patients with airway diseases or relevant cell/animal models, research content involving mucin expression detection, etc.). Duplicate records, non-peer-reviewed literature, studies of low relevance, and those without full-text availability were excluded. A PRISMA 2020 flow diagram illustrating the complete literature search and selection process is provided as Figure 2.

During data synthesis, extraction results from disease cohorts and healthy controls were systematically compared, with analyses conducted on differences across airway disease subtypes. Quantitative comparisons and narrative syntheses identified consistent and divergent findings; contradictions between studies were reconciled through critical evaluation of study design, patient populations, and methodological approaches (Figure 2).

## 3 Results and discussions

### 3.1 The biological basis of MUC5AC/MUC5B

Although MUC5AC and MUC5B are both airway mucins, they differ in morphology, distribution, and function (4). MUC5B is secreted by submucosal glands in the form of strands. MUC5AC is secreted by goblet cells as threads and thin sheets. Due to the high polymorphism of its VNTR region, MUC5AC can generate variants of different lengths, which drive the disease process by altering the rheological properties of mucus (5). Functionally, MUC5AC mediates pathogen capture, while MUC5B serves as the structural basis for mucociliary clearance (6). The dynamic equilibrium between the two is central to maintaining mucus homeostasis, and the disruption of this equilibrium ultimately manifests as the characteristic pathological features of various respiratory diseases (7, 8). In normal/healthy human airways, MUC5B is the dominant secretory mucin in the superficial epithelium and glands, with distal airways being a major site of expression. MUC5B and MUC5AC expression is a property of CCSP-positive secretory cells in superficial airway epithelia (9).

### 3.2 Regulatory pathway

Mucus dysfunction represents a pivotal pathological hallmark common to a spectrum of airway diseases, primarily driven by the dysregulated expression of the gel-forming mucins MUC5AC and MUC5B. Delineating the sophisticated regulatory networks governing these mucins is therefore essential for uncovering underlying disease mechanisms.

The regulatory network controlling MUC5AC and MUC5B expression involves multiple distinct yet interconnected signaling pathways activated by diverse stimuli. Pro-inflammatory cytokines are potent inducers: IL-1β specifically increases the secretion of MUC5AC, but not MUC5B, and alters airway surface liquid (ASL) volume and composition via upregulation of Cl^−^ secretory currents (via CFTR and Ca^2+^-activated Cl^−^ conductance) and inhibition of ENaC (epithelial sodium channel)-mediated Na^+^ absorption (10). Similarly, the chemokine MCP-1 increases both major airway mucins through a cascade initiated by its receptor CCR2B interacting with Gq subunits in caveolae, followed by PLC, PKC, and 44/42 MAPK activation (11). Environmental agents like diesel exhaust particles (DEPs) trigger a broad signaling axis where DEPs activate TLR4, leading to the parallel activation of the MAPK pathway (ERK1/2, p38) and the NF-κB pathway, which jointly upregulate the transcriptional activity of both MUC5AC and MUC5B genes (12). MUC5AC has been associated with type 2 airway inflammation; however, the direct causal relationship between MUC5AC and inflammation mediated through the TLR4/NF-κB pathway requires further validation.

Conversely, therapeutic inhibition of mucin overproduction can target key nodes in these pathways. For instance, wogonoside treatment reduces mucus hypersecretion by downregulating the expression of MUC5AC and the phosphorylation of STAT6 and NF-κB p65, indicating that blocking the NF-κB/STAT6 activation is an effective strategy (13). This ensemble of pathways highlights the complex and stimulus-specific regulation of mucin genes in airway epithelium.

### 3.3 Analysis of disease-specific MUC5AC/MUC5B imbalance patterns

#### 3.3.1 Chronic obstructive pulmonary disease and asthma

Both chronic obstructive pulmonary disease (COPD) and asthma share the core pathological feature of abnormal airway mucus secretion, both conditions are characterized by a disruption in the physiological balance of MUC5AC and MUC5B expression, presenting a characteristic dysregulation pattern of significantly upregulated MUC5AC and relatively insufficient or sluggish MUC5B. This imbalance is closely associated with the onset, progression, severity, acute exacerbations, and treatment response of both diseases, and represents the core mechanism underlying pathological changes in airway mucus.

In the airways of healthy individuals, MUC5B is the primary mucin in the mucus barrier, while MUC5AC plays a complementary functional role through low-level basal expression: MUC5B concentrations can reach 127 nM, approximately eight times that of MUC5AC (16 nM). The MUC5AC/MUC5B ratio is only 0.14. The two maintain a precise homeostatic balance in terms of expression, distribution, concentration, and function (6). Furthermore, in healthy individuals, the concentration of soluble MUC5B in bronchial lavage fluid is more stable and accounts for a higher proportion, while MUC5AC is present only at extremely low levels in a supplementary capacity. The two proteins are precisely regulated through distinct signaling pathways and secretion patterns, jointly ensuring the defensive function of healthy airways without abnormal mucus accumulation (14).

COPD is characterized by mucus hypersecretion driven by a subtype-specific MUC5AC/MUC5B imbalance, with MUC5AC exhibiting a relative fold-change dominance in early-stage disease. A longitudinal analysis of the London COPD Cohort revealed that in individuals with accelerated decline in lung function (an annual decline in FEV_1_ of 156 mL), both MUC5AC and MUC5B concentrations increased significantly during the study period—by 52 and 46.1%, respectively. However, the increase in MUC5AC was markedly greater, leading to a significant rise in the MUC5AC/MUC5B ratio (+63.8%) (15), ultimately resulting in a MUC5AC-dominated pathological imbalance (16). A key distinction exists between simple smoking and established COPD in mucin secretion: smoking alone induces goblet cell MUC5AC upregulation without submucosal gland pathology, while progressive smoking-related airway damage triggers pathological gland hypertrophy in COPD, driving a marked surge in MUC5B secretion (17). Notably, MUC5B is the major mucin in the gel phase of COPD sputum in terms of absolute concentration, a finding validated in large COPD cohorts and consistent with the structural role of MUC5B in mucus gel formation (17). This shift results in a unique COPD mucin profile: MUC5AC dominates in relative expression ratio (bronchial lavage/epithelial tissue), while MUC5B is the predominant structural mucin in sputum gel.

The detailed signaling and transcriptional regulatory mechanisms underlying mucin dysregulation in COPD are described in Section 3.2. Briefly, cigarette smoke serves as a key inducer of MUC5AC upregulation. Epidemiological data reveal a positive correlation between pack-years of smoking and sputum MUC5AC concentration. Rhinoviruses, a common cause of acute exacerbations, can further synergistically induce MUC5AC secretion. These factors act in an intertwined manner, leading to sustained elevation of MUC5AC and delayed MUC5B response, ultimately disrupting the physiological balance between MUC5AC and MUC5B and driving COPD progression (18).

In early disease management, measuring the MUC5AC/MUC5B ratio can help identify high-risk smokers, predict exacerbations, and track disease progression. Therapies aimed specifically at MUC5AC overproduction show considerable promise. For example, EGFR inhibitors have been shown in animal studies to reduce mucus thickness and lower the rate of acute exacerbations (19, 20). N N-acetylcysteine (NAC) reverses cigarette smoke-induced MUC5AC overexpression by inhibiting the ROS/IP3R/Ca^2+^ pathway, providing rationale for combined antioxidant and targeted therapies (21). Smoking cessation significantly reduces MUC5AC levels, while MUC5B expression recovers with prolonged cessation duration.

In asthma, the expression balance between MUC5AC and MUC5B exhibits characteristic dysregulation, characterized by a pattern of significantly upregulated MUC5AC and relatively insufficient MUC5B. This imbalance is closely associated with disease phenotype, severity, and treatment response (22). In patients with mild asthma, induced sputum MUC5AC levels were mucus hypersecretion compared to controls. While the MUC5AC/MUC5B ratio showed an increasing trend, suggesting a relatively higher proportion of MUC5AC (23). As the disease progresses, the MUC5AC positivity rate in airway epithelial goblet cells of moderate-to-severe asthma patients reaches as high as 70–85%, while the MUC5B positivity rate drops to 35–45%. This imbalance correlates positively with asthma severity (24). It is noteworthy that genetic background may further amplify this imbalance. Asthma patients carrying the rs12788104 G allele in the MUC5AC promoter exhibit a 4.6-fold increase in MUC5AC protein secretion compared to non-carriers (25).

The key molecular pathways driving mucin imbalance in asthma are summarized in Section 3.2. Type 2 inflammation serves as the core driver, with IL-4/IL-13 signaling and downstream transcription factors promoting goblet cell metaplasia and MUC5AC overexpression. Neutrophil elastase further reinforces the “inflammation–mucus secretion” vicious cycle. Genetic and epigenetic modifications collectively stabilize this dysregulated state.

This imbalance in mucin expression also provides a clear direction for the clinical diagnosis and management of COPD and asthma: in both conditions, MUC5AC levels and the MUC5AC/MUC5B ratio can serve as biomarkers for screening high-risk populations, monitoring disease progression, and predicting acute exacerbations, offering a more targeted diagnostic value than traditional pulmonary function tests. Therapeutically, both conditions can be addressed by inhibiting excessive MUC5AC secretion and restoring the physiological balance between the two mucins. For example, inhibitors targeting the EGFR pathway can reduce mucus production; animal studies have demonstrated that they can decrease mucus viscosity and reduce the incidence of acute exacerbations (19, 20); The antioxidant N-acetylcysteine can reverse cigarette smoke-induced MUC5AC overexpression by inhibiting the ROS/IP3R/Ca^2+^ pathway (21), providing a theoretical basis for the combined use of antioxidant therapy and targeted therapy. For COPD, smoking cessation should be adopted as a fundamental and cost-effective intervention, as it can significantly reduce MUC5AC levels and gradually restore them to near-normal ranges. Concurrently, MUC5B expression recovers as the duration of smoking cessation increases, suggesting that early smoking cessation helps delay the decline in lung function; For asthma, combined antagonistic drugs targeting key pathways of Type 2 inflammation—such as TSLP and IL-13—can be developed, including novel nanobody-based formulations (26, 27). Anti-IL-13 biologics regulate asthma MUC5AC/MUC5B balance by inhibiting the IL-13-eosinophil degranulation axis, suppressing excessive MUC5AC expression, lowering the abnormally elevated ratio and restoring physiological equilibrium—with superior efficacy in acute fatal asthma featuring MUC5AC-enriched mucus plugs (28). In asthma, high airway TSLP correlates with increased MUC5AC, decreased MUC5B, and elevated imaging mucus plug burden (29); histological analysis confirms MUC5AC-enriched paucigranulocytic plugs (predominant in acute fatal asthma) have a significantly higher MUC5AC/MUC5B ratio. Preclinical and *in vitro* data verify IL-13 induces goblet cell MUC5AC overexpression via the STAT6/SPDEF pathway, while clinical evidence suggests anti-TSLP therapy reduces mucus plug imaging scores, indirectly improving mucin hypersecretion. However, direct evidence from human intervention studies confirming anti-IL-13/TSLP inhibitors directly reduce the airway MUC5AC/MUC5B ratio remains lacking. Concurrently, reliable biomarker detection methods at the protein or genetic level must be established to identify the dominant inflammatory pathways and guide personalized treatment. Ultimately, the treatment of both diseases requires integrating multiple pathways—“anti-inflammatory, mucus-modulating, and maintenance of microenvironmental homeostasis”—and using dynamic monitoring of mucin balance indicators to guide treatment adjustments (6), thereby achieving dynamic regulation of the balance between MUC5AC and MUC5B and developing more precise and effective long-term management plans for patients with COPD and asthma.

#### 3.3.2 Cystic fibrosis

In the pulmonary pathology of cystic fibrosis (CF), the homeostasis imbalance between airway mucins MUC5AC and MUC5B is a core driver of mucus obstruction. This imbalance is not merely a simple increase or decrease in quantity, but rather a complex process involving expression regulation, post-translational modifications, spatial distribution, and dynamic evolution, collectively leading to the formation of pathologic mucus that is difficult to clear.

The most prominent feature of CF airways is the significant elevation in both MUC5AC and MUC5B concentrations, yet their respective increases are disproportionate, resulting in a severely skewed ratio. In fresh-cut airways of newborn CF and non-CF piglets before secondary symptoms, porcine submucosal glands secrete MUC5B, while goblet cells produce MUC5AC as the major mucin with minor MUC5B co-secretion. Morphologically, MUC5B is released from gland ducts as strands of multiple filaments, and MUC5AC from goblet cells as wispy threads or occasional mucin sheets (30). MUC5AC is observed to partially coat MUC5B strands under physiological conditions. Compared with non-CF piglets, MUC5B accumulates more extensively in CF submucosal gland ducts, and MUC5AC sheets deposit on MUC5B strands in CF airway lumens (30). These morphological features of MUC5AC and MUC5B in CF piglet models reveal their distinct structural forms and interactions, indicating differential effects on airway mucociliary transport (30, 31).

Clinical data indicate that MUC5AC and MUC5B concentrations in CF patient sputum can reach 30-fold and 8-fold levels of healthy individuals, respectively. This elevates the MUC5AC/MUC5B ratio from approximately 0.1 in healthy states to 0.4, with this ratio positively correlating with patient age (32). This imbalance was further validated in animal models of CFTR defects: CFTR knockout rats exhibited significantly elevated MUC5B levels by 3 months of age, while MUC5AC levels only markedly increased by 6 months. In newborn CF pigs, the proportion of MUC5AC in airway mucus (0.6) was substantially higher than in wild-type pigs (0.2), and MUC5AC secretion showed a greater increase following carbachol stimulation (30, 31), indicating this is a direct consequence of CFTR loss of function. Notably, newborn CF piglets show early elevated MUC5AC, while progressive human CF involves sequential MUC5B accumulation followed by MUC5AC overexpression. Concurrently, *in vitro* experiments have confirmed that CFTR defects in CF airway epithelial cells result in impaired fluid secretion.

In healthy airways, MUC5AC and MUC5B exhibit distinct secretory roles, a balance disrupted in CF disease. On one hand, widespread metaplasia of goblet cells extends MUC5AC expression from proximal to distal airways. On the other, submucosal glands abnormally begin expressing MUC5AC. Concurrently, structurally altered MUC5B exhibits significantly enhanced adhesion to airway surfaces. Ultimately, MUC5AC and MUC5B form highly adhesive “hybrid gels” throughout the airways, severely impairing mucociliary clearance function. Clearance rates can drop to 40–60% of healthy levels (31, 33).

In cystic fibrosis, dysregulated mucus production is a key mechanism driving disease progression. In human patients and during the progressive stage of established CF disease with secondary pathological manifestations, the pathological process initially involves predominant accumulation of MUC5B, followed by a marked increase in MUC5AC expression as disease severity escalates. These two mucins interact with local inflammatory mediators (such as IL-1β and neutrophil elastase), forming a self-reinforcing feedback loop that collectively increases the risk of mucus plug formation and bacterial colonization (30, 32, 34). Of note, MUC5AC and MUC5B exhibit distinct glycosylation patterns that differentially regulate bacterial adhesion and microbiome composition in CF airways (9). Causative CFTR modulators (e.g., ivacaftor) partially restore mucus homeostasis by reducing total mucin concentration, improving composition ratios, and decreasing mucus adhesiveness. However, these therapies show limited efficacy in advanced patients with extensive airway remodeling, suggesting that early intervention combined with direct mucus-dissolving strategies may be more critical.

In summary, mucus pathology in cystic fibrosis lung disease manifests as a persistent MUC5AC/MUC5B imbalance. MUC5B accumulates first, followed by MUC5AC overexpression, accompanied by extensive alterations in mucin molecular structure, spatial distribution, and function. The ultimate outcome is the formation of highly viscous, difficult-to-clear pathological mucus, constituting the core mechanism of pulmonary pathology in this disease. Therefore, deepening our understanding of and effectively intervening in the MUC5AC/MUC5B imbalance represents a critical research direction for improving the clinical prognosis of cystic fibrosis.

#### 3.3.3 COVID-19

The expression of MUC5AC and MUC5B is dynamically regulated during respiratory disease. Upon viral infection, such as with SARS-CoV-2, their expression is upregulated. Specifically, SARS-CoV-2 induced cytokines (IL-1β, IL-6, and IL-8) and MUC5AC/5B expression through the ACE2 receptor in human nasal epithelial cells (35). Quantitative data from clinical and experimental studies further clarify this dysregulation: in critically ill COVID-19 patients, tracheal aspirates show significantly elevated MUC5AC concentrations (36), while autopsy lung analyses reveal that MUC5B RNA expression is consistently increased across all airway regions, with MUC5AC upregulation being more variable (37). *In vitro* experiments using human bronchial epithelial (HBE) cells infected with SARS-CoV-2 demonstrate that MUC5B and MUC5AC RNA and protein levels peak in the subacute (Day 7) to chronic phase (Day 14) post-infection, with MUC5B protein induction being far more prominent than MUC5AC (37). This induced expression follows a distinct baseline pattern, as MUC5B is constitutively expressed in the healthy airway, whereas MUC5AC is upregulated in response to inflammatory challenge (38). In COVID-19 pathology, airway mucus accumulation has been specifically associated with increased epithelial MUC5B but not MUC5AC expression (37). Beyond gel-forming mucins, transmembrane mucins (e.g., MUC1, MUC16) play a critical protective role in modulating SARS-CoV-2 infection. Human lung epithelial Calu-3 cells and organoid-derived air-liquid interface (ALI) airway cultures express high levels of MUC1 and MUC16 on their apical surfaces, where these mucins colocalize with the ACE2 receptor in close proximity (<40 nm) (39).

During the acute and post-acute (long COVID) phases of COVID-19, there are significant differences in the dynamic changes of the MUC5AC/MUC5B imbalance. In the acute phase, viral replication in HBE cultures peaks on day 3 post-infection and then gradually declines, whereas the induction of mucins—particularly MUC5B—lags behind and persists into the subacute/chronic phase. Clinically, severe acute COVID-19 is characterized by MUC5B-dominant mucus plugging, observed in 90% of severe cases, which directly leads to hypoxia and progressive respiratory failure. These MUC5B-rich mucus plugs undergo altered biophysical properties due to reduced airway fluid secretion and abnormal mucin glycosylation, making them extremely difficult to clear via mucociliary clearance (37). Notably, SARS-CoV-2 infection of human airway epithelial (HAE) cells leads to massive viral shedding (release of large clusters of >200 viral particles in a 2-μm radius) and extensive epithelial cell detachment, processes that are exacerbated by the depletion of intracellular MUC5AC reserves within 2–3 days post-infection. This rapid depletion of MUC5AC may overwhelm the mucosal defense system, thereby facilitating viral transmission (40). In the post-acute (long COVID) phase, persistent mucus hypersecretion remains a key symptom in a substantial proportion of patients (41). Longitudinal studies of post-COVID-19 subjects show sustained upregulation of MUC5B mRNA and potential abnormalities in MUC5AC expression in bronchial biopsies, accompanied by impaired mucociliary clearance and small airway disease (detected via chest CT) (42). This chronic MUC5B-dominant mucin imbalance is associated with persistent cough, dyspnea, and increased risk of secondary bacterial infections (43). Notably, genetic factors such as the MUC5B promoter polymorphism rs35705950 may modulate this mucin imbalance. The minor T allele of rs35705950, a well-established strongest genetic risk factor for idiopathic pulmonary fibrosis (IPF) due to its induction of MUC5B overexpression (44), has been shown to confer protective effects against COVID-19 hospitalization and post-infection pneumonia in individuals of European ancestry. This pleiotropic effect is potentially attributed to regulated MUC5B expression enhancing mucosal host defense against SARS-CoV-2 (45), highlighting that genetic background can shape MUC5AC/MUC5B balance and disease outcomes in viral-induced airway pathology.

Aberrant production of MUC5AC and MUC5B drives clinically significant pathology, primarily through mucus obstruction and impaired host defense. A direct consequence is physical airway blockage, as SARS-CoV-2 infection causes airways to be obstructed by abundant MUC5AC-containing mucus (35). In severe COVID-19, this manifests as MUC5B-dominated mucus plugging observed in 90% of subjects, contributing to hypoxia and inflammation (37). This accumulated mucus creates a nidus for secondary complications, with studies detecting bacterial 16S rRNA signals in mucus plugs of COVID-19 lungs, indicating a high risk of superimposed bacterial infection. Furthermore, specific genetic variations in these mucins are linked to chronic lung disease, as a SNP in MUC5AC increases its tendency to form net-like polymers, showing a significant correlation with idiopathic pulmonary fibrosis (IPF) (38, 46, 47). Beyond obstruction, deficiency or dysfunction of these mucins compromises innate immunity; for instance, Muc5b knockout mice are prone to bacterial infections and exhibit inflammatory infiltrates. Host factors like aging exacerbate these outcomes, as decreased Muc5b production in old mice results in reduced mucociliary clearance, potentially explaining greater viral susceptibility in the elderly (48).

Therapeutic strategies targeting mucin pathology focus on multiple levels. Direct interventions include modulation of key signaling pathways—for instance, inhibiting EGFR/IL-1R (using gefitinib or anakinra) or administering dexamethasone, which reduces MUC5B and MUC5AC expression in SARS-CoV-2-infected HBE cells. A parallel approach involves improving mucus clearance through the development of effective mucolytic agents and strategies to enhance hydration, such as targeting ENaC and CFTR (37). Exploiting the defensive role of mucins, another direction is to boost mucosal immunity by modulating mucin expression or glycosylation—for example, polyphosphate nanoparticles upregulate MUC1 and MUC5AC expression in A549 cells (47). Advancing personalized medicine through the identification of reliable biomarkers (e.g., MUC5B mRNA levels in nasal swabs, MUC5AC protein in serum) is crucial to guide these targeted treatments.

#### 3.3.4 Invasive mucinous adenocarcinoma

Invasive mucinous adenocarcinoma (IMA) is a distinct pathological subtype of lung adenocarcinoma, accounting for approximately 5–10% of all lung adenocarcinomas. Its most prominent pathological feature is the massive accumulation of mucus within tumor cells, forming a characteristic goblet cell morphology. This phenotype is primarily driven by the abnormally high expression of mucins MUC5AC and MUC5B. Rhinoviruses, a common cause of acute exacerbations in COPD, can further synergistically induce MUC5AC secretion in COPD airways.

The synergistic overexpression of MUC5AC and MUC5B in IMA serves as its core molecular hallmark distinguishing it from other lung adenocarcinoma subtypes (49). Studies demonstrate that compared to normal lung tissue and non-mucinous adenocarcinomas (such as squamous-predominant adenocarcinoma, LPA), both mRNA and protein levels of MUC5AC and MUC5B are significantly upregulated in IMA tissue (50). Immunohistochemistry reveals concurrent positivity for both markers in over 70% of IMA cases, with their expression patterns highly overlapping with mucus lakes visualized by Alcian blue staining. Notably, this high expression is closely linked to the molecular background of IMA, particularly in tumors harboring KRAS mutations (present in approximately 40–60% of IMA cases), forming the characteristic IMA molecular phenotype of “KRAS mutation + mucin overexpression.”

The fundamental cause of abnormal mucus accumulation in IMA lies in the dysregulation of the transcriptional regulatory network governing MUC5AC and MUC5B. In addition to the core mucin regulatory pathways described in Section 3.2, IMA is driven by distinct oncogenic and transcriptional programs The key transcription factor SPDEF acts as a master regulator to directly activate MUC5AC and MUC5B transcription. FOXA2 further facilitates mucin expression by modifying chromatin structure at the MUC5AC enhancer. At the oncogenic signaling level, KRAS mutations promote mucin expression via activation of the MEK–ERK pathway, which enhances the activity of FOXA2 and SPDEF. IMA is also frequently characterized by loss of NKX2-1 (TTF-1) expression (51), a transcriptional repressor of mucin genes; its absence relieves inhibition of MUC5AC and MUC5B, resulting in derepressed expression.

Abnormal expression of MUC5AC and MUC5B is closely associated with specific tumor phenotypes and poor clinical outcomes. It primarily induces a mucinous tumor phenotype. In invasive mucinous adenocarcinoma (IMA), the upregulation of MUC5AC is critical for inducing the goblet cell phenotype and, together with transcription factors like HNF4A, defines a gastric-type differentiation program (50, 52, 53). This abnormal mucin secretion directly leads to distinct pathological morphologies, such as intracellular mucin accumulation and mucin lake formation (52). Clinically, these changes correlate with more aggressive disease behavior. Studies show that IMA with high MUC5AC expression has a worse prognosis than TRU-type lung adenocarcinoma (53). Furthermore, specific molecular subtypes carry different prognostic implications; for example, IMA with diffuse MUC6 expression (often co-expressing MUC5AC) may have a relatively favorable outcome, while the KRAS G12D-mutated subtype is associated with shorter relapse-free survival (49, 54). Tumor invasiveness and metastatic potential are also linked to these changes, as evidenced by KRAS-mutated, NKX2-1/TTF1-deleted IMA forming multifocal lesions in the distal bronchiolar epithelium and metastasizing upon loss-of-function TP53 mutation (53).

Therapeutic strategies for MUC5AC/MUC5B-driven lung adenocarcinoma aim to disrupt the core transcriptional programs sustaining mucinous differentiation. Direct targeting of key regulators, such as the HNF4A-MUC5AC axis or SPDEF-mediated enhancer activation of mucin genes, may suppress this phenotype. Restoring the lung lineage factor NKX2-1/TTF1 offers a parallel strategy to repress these oncogenic networks. Future stratification criteria should transcend single driver genes by integrating mucin expression profiles (e.g., MUC5AC/MUC5B vs. MUC1), key transcription factor states (HNF4α, TTF1), and comprehensive genetic variation information. Molecular subtypes defined by this approach will correspond to distinct core-dependent pathways, thereby matching the aforementioned specific therapeutic combinations.

### 3.4 Comparison of models

A common feature across multiple airway diseases is the disruption of the balance between MUC5AC and MUC5B. Although symptoms vary, the specific form of this imbalance is often closely linked to disease progression. Different types of inflammation—whether induced by neutrophils, type 2 immunity, or infection—frequently interfere with normal mucin production through signaling pathways such as EGFR and STAT6. This disruption subsequently weakens the lungs’ self-clearing capacity: excessive MUC5AC renders mucus viscous and difficult to expel, while abnormal MUC5B compromises its structural integrity. These changes collectively create a vicious cycle of mucus hypersecretion, impaired clearance, and recurrent episodes or infections. Notably, alterations in the MUC5AC/MUC5B ratio often correlate with disease severity, indicating this imbalance is an active driver rather than merely a concomitant phenomenon.

The specific form of this imbalance varies by disease. In COPD and asthma, MUC5AC is typically disproportionately elevated (55); cystic fibrosis patients overproduce both mucins, with MUC5B being particularly prominent, resulting in gelatinous mucus (56); in invasive mucinous adenocarcinoma, tumor cells uncontrollably express both mucins simultaneously (49); even in COVID-19 infection, mucin balance shifts, with patterns dynamically evolving from the acute phase to recovery (38). These disease-specific imbalances reveal how distinct etiologies and microenvironments disrupt mucus homeostasis in unique ways.

### 3.5 Analysis of therapeutic targets

Future airway disease therapies are moving from nonspecific mucus clearance toward precision strategies aimed at restoring the physiological balance between MUC5AC and MUC5B, which is essential for normal mucus composition and airway barrier function. Disease-specific interventions for MUC5AC/MUC5B dysregulation are outlined in Table 1. For MUC5AC-dominant hypersecretion in COPD and asthma, treatment focuses on inhibiting overproduction via blockade of IL-13/STAT6, EGFR signaling, or targeting transcriptional regulators such as SPDEF and FOXA2. For MUC5B, context-adaptive strategies apply: preserving its structural function in deficiency states and reducing pathogenic overexpression in cystic fibrosis (CF) through transcriptional inhibition or polymer modification.

**Table 1:** Targeted therapeutic strategies for MUC5AC/MUC5B imbalance in major airway diseases by development stage.

Target disease	Therapeutic strategies (development stage + agent/intervention)	Core mechanism of action
Chronic Obstructive Pulmonary Disease (COPD)	Clinically approved/routine use: N-Acetylcysteine (NAC) (21), Smoking cessation (17); Clinical trial stage: EGFR inhibitors, IL-13/STAT6 pathway inhibitors (13)	1. NAC: Reverse MUC5AC overexpression, mucolysis; lower the abnormally elevated MUC5AC/MUC5B ratio 2. Smoking cessation: Reduce MUC5AC, restore MUC5B; optimize the ratio to physiological level; 3. EGFR/IL-13/STAT6 inhibitors: Inhibit MUC5AC synthesis/transcription; lower the abnormally elevated MUC5AC/MUC5B ratio
Asthma	Clinical trial stage: EGFR inhibitors, IL-13/STAT6 pathway inhibitors (13, 25) Preclinical/conceptual stage: ENaC antagonist + CFTR modulator (combination) (30)	1. EGFR/IL-13/STAT6 inhibitors: Suppress MUC5AC overexpression, block inflammation-mucus cycle; lower the abnormally elevated MUC5AC/MUC5B ratio; 2. ENaC + CFTR modulator: Improve mucus hydration/clearance; optimize the ratio to physiological level
Cystic Fibrosis (CF)	Clinically approved/routine use: Ivacaftor (CFTR modulator) Clinical trial stage: EGFR inhibitors Preclinical/conceptual stage: ENaC antagonist + CFTR modulator (combination) (30)	1. Ivacaftor: Reduce mucin concentration/adhesiveness, improve composition ratio; optimize the ratio to physiological level; 2. EGFR inhibitors: Inhibit MUC5AC/MUC5B abnormal transcription; reduce the excessively skewed MUC5AC/MUC5B ratio; 3. ENaC + CFTR modulator: Alleviate mucus hyperconcentration, enhance clearance; optimize the ratio to physiological level
COVID-19	Clinically approved/routine use: Dexamethasone (37) Clinical trial stage: ACE2 receptor inhibitors (35)	1. Dexamethasone: Anti-inflammation, inhibit MUC5AC/MUC5B overproduction; normalize the dysregulated MUC5AC/MUC5B ratio; 2. ACE2 inhibitors: Block viral invasion, suppress MUC5AC/MUC5B upregulation; prevent the abnormal elevation of MUC5AC/MUC5B ratio
Invasive Mucinous Adenocarcinoma (IMA)	Preclinical/conceptual stage: SPDEF inhibitors, FOXA2 pathway blockers, NKX2-1/TTF1 restoration therapy (51)	1. SPDEF/FOXA2 blockers: Inhibit MUC5AC/MUC5B transcriptional activation; reduce the abnormally high MUC5AC/MUC5B ratio in tumor tissues; 2. NKX2-1/TTF1 restoration: Re-suppress MUC5AC/MUC5B overexpression; restore the ratio to normal lung tissue level

Modulating the physical properties of mucus through next-generation mucolytics or ENaC/CFTR-mediated airway hydration can optimize mucus rheological properties, thereby maintaining the balance of MUC5AC and MUC5B, and thus complementing the aforementioned strategies. In clinical practice, these interventions are embedded in personalized frameworks, where the MUC5AC/MUC5B ratio serves as a biomarker to guide treatment selection and disease stratification. In dynamic diseases like COVID-19, staged therapy is tailored to the evolving imbalance: early focus on epithelial protection and anti-inflammation to prevent disruption; subacute and chronic phases prioritize mucus clearance and homeostatic restoration to reverse dysregulation.

Despite promising preclinical evidence—including *in vitro* studies and animal models—supporting the therapeutic potential of restoring MUC5AC/MUC5B balance, no human clinical trial data are currently available to confirm that such interventions directly improve patient outcomes. This translational gap, from basic research to clinical application, remains a critical barrier that must be addressed before these strategies can be integrated into routine practice. Ultimately, progress in mucus-mediated airway diseases hinges on precision therapies targeting disease-specific MUC5AC/MUC5B imbalances. Restoring and sustaining this balance is key to moving beyond symptomatic management and preserving normal airway function.

## 4 Conclusion

In airway diseases, the state of mucus significantly influences disease progression. This review emphasizes from an alternative perspective that the dynamic equilibrium between MUC5AC and MUC5B is crucial for maintaining airway health. When disrupted, this balance gives rise to disease-characteristic patterns of imbalance, forming a core component of the pathophysiology underlying multiple airway disorders. From the excessive dominance of MUC5AC in COPD and asthma to the abnormal drive of MUC5B in cystic fibrosis (CF), these imbalances collectively lead to impaired mucus clearance, persistent inflammation, and recurrent infections, thereby continuously propelling disease progression.

In summary, research on MUC5AC/MUC5B imbalance holds promise not only for advancing the development of personalized biomarkers to support early identification and stratified treatment, but also for guiding novel therapeutic strategies targeting mucus synthesis regulation and restoring mucus clearance function. From elucidating the mechanisms of imbalance to implementing balance restoration, this approach may open new avenues for precision intervention and long-term management of airway diseases.

## 5 Innovation and outlook

In previous studies, mucus research on airway diseases often measured only single mucin levels or total mucus volume. This approach struggles to distinguish disease-specific pathological mechanisms. This paper proposes a more comprehensive model: MUC5AC and MUC5B function as a synergistic axis, critical for maintaining airway homeostasis. Most airway diseases can be understood as specific disruptions of this balance.

From this perspective, airway health depends less on the absolute content of a single mucin and more on the functional balance between the two—MUC5AC participates in particle trapping, while MUC5B provides the structural foundation for clearance. When specific triggers (such as cigarette smoke, viral infection, or genetic susceptibility) disrupt this equilibrium through particular pathways (e.g., EGFR, IL-13, or IL-17 signaling), disease ensues, ultimately manifesting as distinct clinical and pathological phenotypes.

A central insight emerging from this review lies in its comparative analysis of MUC5AC/MUC5B imbalance across airway diseases. Rather than merely noting the predominance of MUC5AC in COPD and asthma, we delineate how these mucins operate differently in other conditions. Placing these varied expressions within a common framework reveals a shared pathogenic theme: the loss of mucin equilibrium. Accordingly, future treatments for mucus dysfunction should move beyond nonsuppressive strategies. Instead, they might adopt a precision approach—first clarifying the disease-specific drivers of imbalance, then targeting the restoration of physiological mucin balance as a therapeutic priority.

## References

[1] Livraghi-Butrico A Grubb BR Wilkinson KJ Volmer AS Burns KA Evans CM . Contribution of mucus concentration and secreted mucins Muc5ac and Muc5b to the pathogenesis of muco-obstructive lung disease. Mucosal Immunol. (2017) 10:395–407. doi: 10.1038/mi.2016.63, 27435107 PMC5250616

[2] Song D Iverson E Kaler L Boboltz A Scull MA Duncan GA. MUC5B mobilizes and MUC5AC spatially aligns mucociliary transport on human airway epithelium. Sci Adv. (2022) 8:eabq5049. doi: 10.1126/sciadv.abq5049, 36427316 PMC9699686

[3] Symmes BA Stefanski AL Magin CM Evans CM. Role of mucins in lung homeostasis: regulated expression and biosynthesis in health and disease. Biochem Soc Trans. (2018) 46:707–19. doi: 10.1042/BST20170455, 29802217 PMC8359647

[4] Ostedgaard LS Moninger TO McMenimen JD Sawin NM Parker CP Thornell IM . Gel-forming mucins form distinct morphologic structures in airways. Proc Natl Acad Sci USA. (2017) 114:6842–7. doi: 10.1073/pnas.1703228114, 28607090 PMC5495256

[5] Plender EG Prodanov T Hsieh PH Nizamis E Harvey WT Sulovari A . Structural and genetic diversity in the secreted mucins MUC5AC and MUC5B. Am J Hum Genet. (2024) 111:1700–16. doi: 10.1016/j.ajhg.2024.06.007, 38991590 PMC11344006

[6] Pincikova T Merikallio H Kotortsi I Karimi R Li CX Lappi-Blanco E . Expression levels of MUC5AC and MUC5B in airway goblet cells are associated with traits of COPD and progression of chronic airflow limitation. Int J Mol Sci. (2024) 25:1365. doi: 10.3390/ijms252413653, 39769414 PMC11678853

[7] Kesimer M. Mucins MUC5AC and MUC5B in the airways: MUCing around together. Am J Respir Crit Care Med. (2022) 206:1055–7. doi: 10.1164/rccm.202208-1459ED, 35938865 PMC9704829

[8] Jaramillo AM Vladar EK Holguin F Dickey BF Evans CM. Emerging cell and molecular targets for treating mucus hypersecretion in asthma. Allergol Int. (2024) 73:375–81. doi: 10.1016/j.alit.2024.04.002, 38692992 PMC11491148

[9] Okuda K Chen G Subramani DB Wolf M Gilmore RC Kato T . Localization of secretory mucins MUC5AC and MUC5B in Normal/healthy human airways. Am J Respir Crit Care Med. (2019) 199:715–27. doi: 10.1164/rccm.201804-0734OC, 30352166 PMC6423099

[10] Gray T Coakley R Hirsh A Thornton D Kirkham S Koo JS . Regulation of MUC5AC mucin secretion and airway surface liquid metabolism by IL-1beta in human bronchial epithelia. Am J Physiol Lung Cell Mol Physiol. (2004) 286:L320–30. doi: 10.1152/ajplung.00440.2002, 14527933

[11] Monzon ME Forteza RM Casalino-Matsuda SM. MCP-1/CCR2B-dependent loop upregulates MUC5AC and MUC5B in human airway epithelium. Am J Physiol Lung Cell Mol Physiol. (2011) 300:L204–15. doi: 10.1152/ajplung.00292.2010, 21097527 PMC3043814

[12] Na HG Kim YD Choi YS Bae CH Song SY. Diesel exhaust particles elevate MUC5AC and MUC5B expression via the TLR4-mediated activation of ERK1/2, p38 MAPK, and NF-κB signaling pathways in human airway epithelial cells. Biochem Biophys Res Commun. (2019) 512:53–9. doi: 10.1016/j.bbrc.2019.02.146, 30857636

[13] Yu X Cai B Yu L Li N Wu C Hu Z . Wogonoside ameliorates airway inflammation and mucus hypersecretion via NF-κB/STAT6 Signaling in ovalbumin-induced murine acute asthma. J Agric Food Chem. (2024) 72:7033–42. doi: 10.1021/acs.jafc.3c04082, 38507725

[14] Ye Q Opoku G Orlov M Jaramillo AM Holguin F Vladar EK . Mucins and their roles in asthma. Immunol Rev. (2025) 331:e70034. doi: 10.1111/imr.70034, 40305069 PMC12643232

[15] Meldrum OW Donaldson GC Narayana JK Ivan FX Jaggi TK Mac Aogáin M . Accelerated lung function decline and mucus-microbe evolution in chronic obstructive pulmonary disease. Am J Respir Crit Care Med. (2024) 210:298–310. doi: 10.1164/rccm.202306-1060OC, 38315959 PMC11348959

[16] Radicioni G Ceppe A Ford AA Alexis NE Barr RG Bleecker ER . Airway mucin MUC5AC and MUC5B concentrations and the initiation and progression of chronic obstructive pulmonary disease: an analysis of the SPIROMICS cohort. Lancet Respir Med. (2021) 9:1241–54. doi: 10.1016/S2213-2600(21)00079-5, 34058148 PMC8570975

[17] Kirkham S Kolsum U Rousseau K Singh D Vestbo J Thornton DJ. MUC5B is the major mucin in the gel phase of sputum in chronic obstructive pulmonary disease. Am J Respir Crit Care Med. (2008) 178:1033–9. doi: 10.1164/rccm.200803-391OC, 18776153 PMC2643221

[18] Singanayagam A Footitt J Marczynski M Radicioni G Cross MT Finney LJ . Airway mucins promote immunopathology in virus-exacerbated chronic obstructive pulmonary disease. J Clin Invest. (2022) 132:e120901. doi: 10.1172/JCI120901, 35239513 PMC9012283

[19] Balkrishna A Solleti SK Singh H Verma S Sharma N Nain P . Herbal decoction Divya-Swasari-Kwath attenuates airway inflammation and remodeling through Nrf-2 mediated antioxidant lung defence in mouse model of allergic asthma. Phytomedicine. (2020) 78:153295. doi: 10.1016/j.phymed.2020.153295, 32795904

[20] Yamamoto A Sly PD Khachatryan L Begum N Yeo AJ Robinson PD . Astaxanthin protects against environmentally persistent free radical-induced oxidative stress in well-differentiated respiratory epithelium. Redox Biol. (2025) 81:103542. doi: 10.1016/j.redox.2025.103542 39952200 PMC11875192

[21] Wu XJ Zhang GY Du XZ. Cigarette smoke extract induces MUC5AC expression through the ROS/ IP3R/Ca2+pathway in Calu-3 cells. Int J Chron Obstruct Pulmon Dis. (2024) 19:1635–47. doi: 10.2147/COPD.S469866, 39045541 PMC11264152

[22] Li X Li H Christenson SA Castro M Denlinger LC Erzurum SC . Genetic analyses of chr11p15.5 region identify MUC5AC-MUC5B associated with asthma-related phenotypes. J Asthma. (2023) 60:1824–35. doi: 10.1080/02770903.2023.2193631, 36946148 PMC10524756

[23] Tajiri T Matsumoto H Jinnai M Kanemitsu Y Nagasaki T Iwata T . Pathophysiological relevance of sputum MUC5AC and MUC5B levels in patients with mild asthma. Allergol Int. (2022) 71:193–9. doi: 10.1016/j.alit.2021.09.003, 34656442

[24] Li X Guerra S Ledford JG Kraft M Li H Hastie AT . Low CC16 mRNA expression levels in bronchial epithelial cells are associated with asthma severity. Am J Respir Crit Care Med. (2023) 207:438–51. doi: 10.1164/rccm.202206-1230OC, 36066606 PMC9940145

[25] Sajuthi SP Everman JL Jackson ND Saef B Rios CL Moore CM . Nasal airway transcriptome-wide association study of asthma reveals genetically driven mucus pathobiology. Nat Commun. (2022) 13:1632. doi: 10.1038/s41467-022-28973-7, 35347136 PMC8960819

[26] Venegas Garrido C Mukherjee M Svenningsen S Nair P. Eosinophil-mucus interplay in severe asthma: implications for treatment with biologicals. Allergol Int. (2024) 73:351–61. doi: 10.1016/j.alit.2024.03.001, 38485545

[27] Alharris E Mohammed A Alghetaa H Zhou J Nagarkatti M Nagarkatti P. The ability of resveratrol to attenuate ovalbumin-mediated allergic asthma is associated with changes in microbiota involving the gut-lung axis, enhanced barrier function and decreased inflammation in the lungs. Front Immunol. (2022) 13:805770. doi: 10.3389/fimmu.2022.805770, 35265071 PMC8898895

[28] Liegeois MA Hsieh A al-Fouadi M Charbit AR Yang CX Hackett TL . Cellular and molecular features of asthma mucus plugs provide clues about their formation and persistence. J Clin Invest. (2025) 135:e186889. doi: 10.1172/JCI186889, 40091838 PMC11910225

[29] Khanna K Tang M Jackson ND Johansson MW Bleecker ER Castro M . High airway thymic stromal lymphopoietin in asthma is associated with type 2 inflammation, mucus plugging, and airway remodeling. J Allergy Clin Immunol. (2026) 157:616–26. doi: 10.1016/j.jaci.2025.11.014, 41397482

[30] Keith JD Henderson AG Fernandez-Petty CM Davis JM Oden AM Birket SE. Muc5b contributes to mucus abnormality in rat models of cystic fibrosis. Front Physiol. (2022) 13:884166. doi: 10.3389/fphys.2022.884166, 35574458 PMC9096080

[31] Rodriguez-Piñeiro AM Jaudas F Klymiuk N Bähr A Hansson GC Ermund A. Proteome of airway surface liquid and mucus in newborn wildtype and cystic fibrosis piglets. Respir Res. (2023) 24:83. doi: 10.1186/s12931-023-02381-x, 36927357 PMC10022022

[32] Batson BD Zorn BT Radicioni G Livengood SS Kumagai T Dang H . Cystic fibrosis airway mucus Hyperconcentration produces a vicious cycle of mucin, pathogen, and inflammatory interactions that promotes disease persistence. Am J Respir Cell Mol Biol. (2022) 67:253–65. doi: 10.1165/rcmb.2021-0359OC, 35486871 PMC9348562

[33] Abdullah LH Coakley R Webster MJ Zhu Y Tarran R Radicioni G . Mucin production and hydration responses to mucopurulent materials in Normal versus cystic fibrosis airway epithelia. Am J Respir Crit Care Med. (2018) 197:481–91. doi: 10.1164/rccm.201706-1139OC, 29099608 PMC5821906

[34] Chen G Sun L Kato T Okuda K Martino MB Abzhanova A . IL-1β dominates the promucin secretory cytokine profile in cystic fibrosis. J Clin Invest. (2019) 129:4433–50. doi: 10.1172/JCI125669, 31524632 PMC6763234

[35] Lee S Na HG Choi YS Bae CH Song SY Kim YD. SARS-CoV-2 induces expression of cytokine and MUC5AC/5B in human nasal epithelial cell through ACE 2 receptor. Biomed Res Int. (2022) 2022:2743046. doi: 10.1155/2022/2743046, 35692597 PMC9184209

[36] Lu W Liu X Wang T Liu F Zhu A Lin Y . Elevated MUC1 and MUC5AC mucin protein levels in airway mucus of critical ill COVID-19 patients. J Med Virol. (2021) 93:582–4. doi: 10.1002/jmv.26406, 32776556 PMC7436726

[37] Kato T Asakura T Edwards CE Dang H Mikami Y Okuda K . Prevalence and mechanisms of mucus accumulation in COVID-19 lung disease. Am J Respir Crit Care Med. (2022) 206:1336–52. doi: 10.1164/rccm.202111-2606OC, 35816430 PMC9746856

[38] Chatterjee M van Putten JPM Strijbis K. Defensive properties of mucin glycoproteins during respiratory infections—relevance for SARS-CoV-2. MBio. (2020) 11:e02374–20. doi: 10.1128/mBio.02374-20, 33184103 PMC7663010

[39] Chatterjee M Huang LZX Mykytyn AZ Wang C Lamers MM Westendorp B . Glycosylated extracellular mucin domains protect against SARS-CoV-2 infection at the respiratory surface. PLoS Pathog. (2023) 19:e1011571. doi: 10.1371/journal.ppat.1011571, 37561789 PMC10464970

[40] Morrison CB Edwards CE Shaffer KM Araba KC Wykoff JA Williams DR . SARS-CoV-2 infection of airway cells causes intense viral and cell shedding, two spreading mechanisms affected by IL-13. Proc Natl Acad Sci USA. (2022) 119:e2119680119. doi: 10.1073/pnas.2119680119, 35353667 PMC9169748

[41] Manckoundia P Franon E. Is persistent thick copious mucus a long-term symptom of COVID-19? Eur J Case Rep Intern Med. (2020) 7:002145. doi: 10.12890/2020_002145, 33457378 PMC7806295

[42] Cho JL Villacreses R Nagpal P Guo J Pezzulo AA Thurman AL . Quantitative chest CT assessment of small airways disease in post-acute SARS-CoV-2 infection. Radiology. (2022) 304:185–92. doi: 10.1148/radiol.212170, 35289657 PMC9270680

[43] Manna S Baindara P Mandal SM. Molecular pathogenesis of secondary bacterial infection associated to viral infections including SARS-CoV-2. J Infect Public Health. (2020) 13:1397–404. doi: 10.1016/j.jiph.2020.07.003, 32712106 PMC7359806

[44] Biondini D Cocconcelli E Bernardinello N Lorenzoni G Rigobello C Lococo S . Prognostic role of MUC5B rs35705950 genotype in patients with idiopathic pulmonary fibrosis (IPF) on antifibrotic treatment. Respir Res. (2021) 22:98. doi: 10.1186/s12931-021-01694-z, 33794872 PMC8017848

[45] Verma A Minnier J Wan ES Huffman JE Gao L Joseph J . A MUC5B gene polymorphism, rs35705950-T, confers protective effects against COVID-19 hospitalization but not severe disease or mortality. Am J Respir Crit Care Med. (2022) 206:1220–9. doi: 10.1164/rccm.202109-2166OC, 35771531 PMC9746845

[46] Yin W Cao W Zhou G Wang L Sun J Zhu A . Analysis of pathological changes in the epithelium in COVID-19 patient airways. ERJ Open Res. (2021) 7:00690–2020. doi: 10.1183/23120541.00690-2020, 33829055 PMC7898030

[47] Schepler H Wang X Neufurth M Wang S Schröder HC Müller WEG. The therapeutic potential of inorganic polyphosphate: a versatile physiological polymer to control coronavirus disease (COVID-19). Theranostics. (2021) 11:6193–213. doi: 10.7150/thno.59535, 33995653 PMC8120197

[48] Roy MG Livraghi-Butrico A Fletcher AA McElwee MM Evans SE Boerner RM . Muc5b is required for airway defence. Nature. (2014) 505:412–6. doi: 10.1038/nature12807, 24317696 PMC4001806

[49] Duruisseaux M Antoine M Rabbe N Rodenas A Mc Leer-Florin A Lacave R . Lepidic predominant adenocarcinoma and invasive mucinous adenocarcinoma of the lung exhibit specific mucin expression in relation with oncogenic drivers. Lung Cancer. (2017) 109:92–100. doi: 10.1016/j.lungcan.2017.05.007, 28577958

[50] Guo M Tomoshige K Meister M Muley T Fukazawa T Tsuchiya T . Gene signature driving invasive mucinous adenocarcinoma of the lung. EMBO Mol Med. (2017) 9:462–81. doi: 10.15252/emmm.201606711, 28255028 PMC5376761

[51] Tomoshige K Stuart WD Fink-Baldauf IM Ito M Tsuchiya T Nagayasu T . FOXA2 cooperates with mutant KRAS to drive invasive mucinous adenocarcinoma of the lung. Cancer Res. (2023) 83:1443–58. doi: 10.1158/0008-5472.CAN-22-2805, 37067057 PMC10160002

[52] Sonzogni A Bianchi F Fabbri A Cossa M Rossi G Cavazza A . Pulmonary adenocarcinoma with mucin production modulates phenotype according to common genetic traits: a reappraisal of mucinous adenocarcinoma and colloid adenocarcinoma. J Pathol Clin Res. (2017) 3:139–51. doi: 10.1002/cjp2.67, 28451462 PMC5402180

[53] Koh MJ Shin DH Lee SJ Hwang CS Lee HJ Kim A . Gastric-type gene expression and phenotype in non-terminal respiratory unit type adenocarcinoma of the lung with invasive mucinous adenocarcinoma morphology. Histopathology. (2020) 76:898–905. doi: 10.1111/his.14077, 31985086

[54] Kishikawa S Hayashi T Saito T Takamochi K Kohsaka S Sano K . Diffuse expression of MUC6 defines a distinct clinicopathological subset of pulmonary invasive mucinous adenocarcinoma. Mod Pathol. (2021) 34:786–97. doi: 10.1038/s41379-020-00690-w, 33024306

[55] Bonser L Erle D. Airway mucus and asthma: the role of MUC5AC and MUC5B. J Clin Med. (2017) 112. doi: 10.3390/jcm6120112 PMC5742801 29186064

[56] Martínez-Antón A DeBolós C Garrido M Roca-Ferrer J Barranco C Alobid I . Mucin genes have different expression patterns in healthy and diseased upper airway mucosa. Clin Exp Allergy. (2006) 36:448–57. doi: 10.1111/j.1365-2222.2006.02451.x, 16630149

